# MRI-compatible voltage-based electroanatomic mapping system for 3T MR-guided cardiac electrophysiology: swine validations

**DOI:** 10.1186/1532-429X-16-S1-P140

**Published:** 2014-01-16

**Authors:** Shelley H Zhang, Zion T Tse, Charles L Dumoulin, Israel Bryd, Jeffrey Schweitzer, Ronald G Watkins, Kim Butts-Pauly, Raymond Y Kwong, Chirag R Barbhaiya, William G Stevenson, Ferenc Jolesz, Ehud J Schmidt

**Affiliations:** 1 Brigham and Women's Hospital, Boston, Massachusetts, USA; 2 Engineering, The University of Georgia, Athens, Georgia, USA; 3 Radiology, Cincinnati Children's Hospital Medical Center, Cincinnati, Ohio, USA; 4 Cardiovascular and Ablation Technologies Division, St Jude Medical Inc, St. Paul, Minnesota, USA; 5 Radiology, Stanford University, Stanford, California, USA

## Background

MRI provides luminal, edema, & scar images which assist in the Electrophysiological (EP) treatment of ventricular and atrial arrhythmias]. Until a complete set of MR-compatible EP-devices is available, patients must be repeatedly moved between the MRI, where imaging and mapping occur, to the conventional EP suite, where puncture, navigation and Radio-Frequency Ablation occur. MRI-conditional voltage-based electroanatomic mapping (EAM) would permit efficient MRI-guided EP, with registration-free continuation outside MRI, utilizing X-ray, Intra-Cardiac-Echo (ICE) and EAM guidance. A 1.5T EnSite™ Velocity™ cardiac mapping system, a voltage-based EAM available from St. Jude Medical was previously validated [1,2]. Multiple-catheter EAM (localization and intra-cardiac electrocardiogram (EGM) measurement) inside a 3T MRI requires modifications.

## Methods

**Figure 1:**
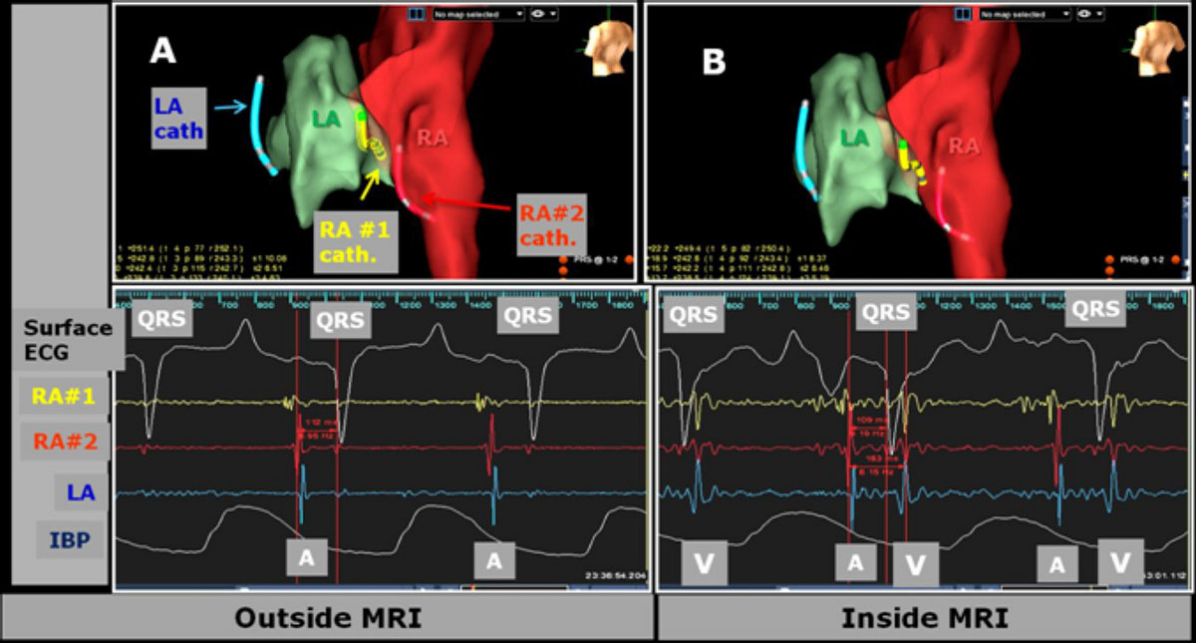
**EAM maps acquired (A) outside MRI versus (B) inside the 3T MRI**. Three simultaneously navigated catheters (LA, RA#1 and RA#2) are shown. Upper images show EAM maps. Lower images show surface ECG, intra-cardiac EGM (RA#1, RA#2 and LA catheter tip electrodes), as well as intra-aortic IBP traces. QRS denotes start of ECG cycle. V and A denote Ventricular and Arterial signals, respectively. A strong superimposed ventricular signal was observed on all atrial intra-cardiac ECG traces (extreme-right red vertical line in (B)) acquired inside the MRI, a result of the strong 3T Magnetohydrodynamic voltage.

An EnSite™ Velocity™ system was connected to an electronic switching circuit that prevents induced MRI gradient-ramp noise from corrupting ECG fidelity. Electrode tracking is preserved with software blanking. The system also included RF-filtered electrical lines, modified EnSite™ NavX™surface electrodes, and gold electrode EP catheters [2]. Trans-septal punctures were made in 2 intubated swine under X-ray & ICE guidance. Millar catheters were placed in the aorta to monitor Invasive Blood Pressure (IBP). The swine were moved to a Siemens 3.0T MRI suite equipped with an EnSite™ Velocity™ system. A 12-lead MRI-compatible ECG [3] was also used. Three EP catheters, with 4 voltage-tracked electrodes each, were navigated simultaneously inside the MRI to acquire EAM of the heart's left & right sides, with a coronary sinus catheter for physiological reference. Imaging & EAM were performed simultaneously (Figure 1). To measure the EnSite™ Velocity™ system's catheter tracking accuracy during MR imaging, catheters were navigated to specific anatomic regions, and the change in location was observed during imaging over 10 sec increments.

## Results

**Figure 2:**
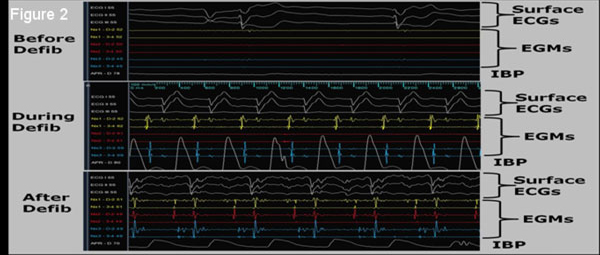
**Surface ECGs, EGMs (Intra-cardiac ECGs) and IBP traces taken before, during, and after successful resuscitation (compression and defibrillation) of a cardiac event which occurred inside the MRI**. The cardiac event was recorded during mapping of the base of the swine's Left Atrium.

**Figure 3:**
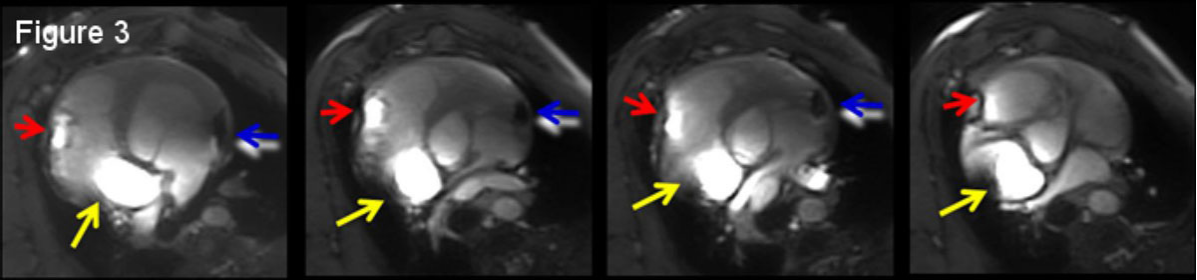
**The three EP catheters (LA, RA#1 and RA#2) were passively detected in multi-slice SSFP cine images as a result of over-tipping (red and yellow arrows) on the catheter shaft as well as susceptibility artifacts (blue arrows) at the tip**.

EAM & catheter navigation in swine were performed both inside & outside the MRI at >20 frames-per-second without re-registration (Figure 1). Imaging was conducted simultaneously with tracking, with catheter position stable (+1 mm) during imaging in TR>32 ms sequences. Median catheter electrode locations changed by < 0.5 mm between inside and outside MRI. An added ventricular signal was observed on intra-MRI atrial EGMs (Figure 1B), due to Magnetohydrodynamics. IBP detected a cardiac event (Figure 2), followed by successful CPR & defibrillation in the MRI suite. MRI Image quality reduction was < 5%, although catheter artifacts during SSFP were excessive (Figure 3), requiring catheter redesign.

## Conclusions

3T MRI-conditional voltage tracking allows simultaneous catheter tracking & MR imaging, permitting registration-free EAM inside & outside MRI during EP procedures.

## Funding

NIH U41-RR019703 & R03-EB013873-01A1, AHA 10SDG261039.
